# Comparative transcriptional analyses of preclinical models and patient samples reveal MYC and RELA driven expression patterns that define the molecular landscape of IBC

**DOI:** 10.1038/s41523-021-00379-6

**Published:** 2022-01-18

**Authors:** Charlotte Rypens, François Bertucci, Pascal Finetti, Fredika Robertson, Sandra V. Fernandez, Naoto Ueno, Wendy A. Woodward, Kenneth Van Golen, Peter Vermeulen, Luc Dirix, Patrice Viens, Daniel Birnbaum, Gayathri R. Devi, Massimo Cristofanilli, Steven Van Laere

**Affiliations:** 1 Translational Cancer Research Unit, GZA Hospitals Sint-Augustinus, Antwerp, Belgium; 2 Center for Oncological Research (CORE), Integrated Personalized and Precision Oncology Network (IPPON), University of Antwerp, Marseille, France; 3 Predictive Oncology laboratory, Centre de Recherche en Cancérologie de Marseille (CRCM), Institut Paoli-Calmettes, INSERM UMR1068, CNRS UMR725, Aix-Marseille Université, Marseille, France; 4 Department of Medical Oncology, CRCM, Institut Paoli-Calmettes, Marseille, France; 5 University of Texas M.D. Anderson Cancer Center, Houston, TX USA; 6 Fox Chase Cancer Center, Philadelphia, PA USA; 7 MD Anderson Morgan Welch Inflammatory Breast Cancer Research Program and Clinic, The University of Texas MD Anderson Cancer Center, Houston, TX USA; 8 Department of Radiation Oncology, The University of Texas MD Anderson Cancer Center, Houston, TX USA; 9 Biological Sciences, University of Delaware, Newark, DE USA; 10 Department of Surgery, Division of Surgical Sciences, Duke University School of Medicine, Durham, NC USA; 11 Duke Consortium for Inflammatory Breast Cancer, Duke Cancer Institute, Durham, NC UK; 12 Department of Medicine, Division of Hematology and Oncology, Robert H Lurie Comprehensive Cancer Center, Northwestern University, Chicago, IL USA

**Keywords:** Cancer models, Breast cancer

## Abstract

Inflammatory breast cancer (IBC) is an aggressive disease for which the spectrum of preclinical models was rather limited in the past. More recently, novel cell lines and xenografts have been developed. This study evaluates the transcriptome of an extended series of IBC preclinical models and performed a comparative analysis with patient samples to determine the extent to which the current models recapitulate the molecular characteristics of IBC observed clinically. We demonstrate that the IBC preclinical models are exclusively estrogen receptor (ER)-negative and of the basal-like subtype, which reflects to some extent the predominance of these subtypes in patient samples. The IBC-specific 79-signature we previously reported was retrained and discriminated between IBC and non-IBC preclinical models, but with a relatively high rate of false positive predictions. Further analyses of gene expression profiles revealed important roles for cell proliferation, MYC transcriptional activity, and TNFɑ/NFκB in the biology of IBC. Patterns of MYC expression and transcriptional activity were further explored in patient samples, which revealed interactions with ESR1 expression that are contrasting in IBC and nIBC and notable given the comparatively poor outcomes of ER+ IBC. Our analyses also suggest important roles for NMYC, MXD3, MAX, and MLX in shaping MYC signaling in IBC. Overall, we demonstrate that the IBC preclinical models can be used to unravel cancer cell intrinsic molecular features, and thus constitute valuable research tools. Nevertheless, the current lack of ER-positive IBC models remains a major hurdle, particularly since interactions with the ER pathway appear to be relevant for IBC.

## Introduction

Inflammatory breast cancer (IBC) is an aggressive and highly metastatic form of breast cancer. At the time of initial diagnosis, virtually all patients have lymph node involvement and 30% present with distant metastases^1^. As a consequence of the rapid onset and early metastasis, patients with IBC display an unfavorable prognosis, with 5-year overall survival rates of 40% despite multimodality treatment^2–4^. IBC is a clinical diagnosis based on the rapid onset of inflammatory symptoms: patients present with a red, enlarged breast associated with shooting pains and warmth. In addition, skin changes (e.g., “*peau d’orange*”) and nipple retraction are often observed and typically, no palpable tumor mass is present^5–7^.

In 2008, the Inflammatory Breast Cancer-International Consortium (IBC-IC) was established by investigators in this field, with the ultimate aim of accelerating IBC research. The compelling need for this alignment of researches was based on the fact that despite many efforts over decades of research, IBC remained a poorly characterized disease void of specific targets for molecular therapy^8^. The need for better, more efficient, and IBC-specific treatment options is underscored by the fact that there are no significant changes in overall survival of patients up till now. In addition, IBC can be regarded as a human model for aggressive (breast) cancer behavior in general.

The first project of the IBC-IC involved the identification of a molecular profile of IBC using a large multicentric series of clinical samples. A set of 79 probe sets with an IBC-specific and molecular subtype-independent gene expression profile was identified and validated. Translating the IBC signature into pathways and processes indicated that alterations in TGFβ signaling may be an important driver^9^, which is confirmed in a more recent study^10^. In addition, a molecular signature predicting pathological complete response to neoadjuvant chemotherapy in IBC was identified^11^ and catalogs of genomic alterations were described^12^. In parallel, the role of the tumor microenvironment (TME) in IBC development and progression has been also increasingly emphasized^13–18^. Additionally, efforts were also focused on developing greater numbers of preclinical IBC models of different molecular subtypes, allowing researchers to perform functional validations in more versatile genetic backgrounds. Traditionally, five preclinical models have been used for IBC research: three established cell lines (i.e., KPL4, SUM149, and SUM190) and two xenograft models (i.e., Mary-X and WIBC9). The preclinical models of IBC are either triple negative or HER2-amplified, which is reflective of the most prevalent subtypes of this disease^19–30^. Within the last years, novel IBC models have been generated amongst others by researchers at the Fox Chase Cancer Center (i.e., FC-IBC-01 and FC-IBC-02), The University of Texas MD Anderson Cancer Center (i.e., MDA-IBC-03), the Thomas Jefferson University (i.e., TJ-IBC-04 and TJ-IBC-09), and the GZA Hospital Sint-Augustinus (i.e., UA-IBC-01)^31^.

However, the complete molecular characterization and comparative analyses of these cell lines remains to be completed. Therefore, we report here a comprehensive analysis of gene expression data from IBC and non-IBC (nIBC) preclinical models and patient samples. Our primary goal was to gain insight into the molecular characteristics of the above-described IBC preclinical models and to identify features also exhibited by IBC cells in human tissue samples. This set of features will be crucial knowledge when setting up functional validation experiments for data reported in patient samples. In addition, to broaden the clinical perspectives of this panel of IBC preclinical models, their sensitivity profile to a wide range of therapeutic agents was estimated using the CMap data set of 1.3 million L1000 signatures that reflect transcriptional responses of human cells to chemical and genetic perturbations. It stands to reason that these efforts will also contribute to a more detailed comprehension of biological themes intrinsic to IBC cells.

## Results

### Cluster analysis and molecular subtyping

**Fig. 1:**
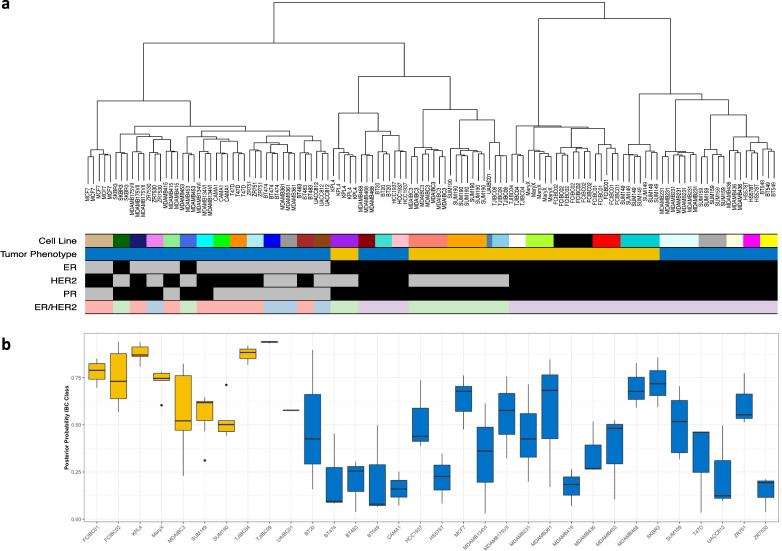
Molecular characterization and classification of (inflammatory) breast cancer cell lines. **a** Dendrogram resulting from an unsupervised hierarchical cluster analysis performed on the normalized expression data set of preclinical models prior to averaging. The different cell lines are indicated using different colors in the annotation track underneath the dendrogram, in addition to the tumor phenotype (blue = nIBC; yellow = IBC), the ER status (gray = ER+; black = ER−), the HER2 status (gray = HER2−; black = HER2+), the PR status (gray = PR+; black = PR−), and the ER/HER2 combined subtypes (red = ER+/HER2−; green = ER−/HER2+; blue = ER+/HER2+; purple = ER−/HER2−). **b** The classification scores of the preclinical models are shown in boxplot format. The different preclinical models are shown along the X-axis and the Y-axis represents the posterior probability scores resulting from applying the IBC classification models. Boxes are color-coded according to the tumor phenotype: blue = nIBC and yellow = IBC.

To investigate differences between IBC (*n* = 10) and nIBC (*n* = 22) preclinical models, we merged expression profile analysis performed in our Institution with external gene expression data from various public resources (Gene Expression Omnibus: GSE12777, GSE16795, and GSE40464; and ArrayExpress: E-MTAB-7). To assess the efficiency of the normalization strategy, unsupervised hierarchical clustering analysis (UHCA) was performed for all 124 profiles and for the 500 most variable genes selected by standard deviation. Results are shown in Fig. 1a. The NbClust algorithm identified four clusters in the data set that were significantly associated with the ER status (*P* < 0.001), the PR status (*P* < 0.001), the HER2 status (*P* = 0.043), the ER/HER2 combined subtypes (*P* < 0.001), and the IBC/nIBC tumor phenotype (*P* < 0.001). Using multinomial regression analyses, we demonstrated that the ER/HER2 combined subtypes were the best predictor of the clustering pattern (AIC = 59.881), followed by the ER status (AIC = 63.195), the tumor phenotype (AIC = 70.293), the PR status (AIC = 74.277), and the HER2 status (AIC = 82.575). A multivariate model containing the tumor phenotype and the ER/HER2 combined subtypes (AIC = 45.589) was significantly better in predicting the clustering pattern as compared to the ER/HER2 combined subtypes alone (Likelihood ratio test; *P* < 0.001). Addition of the PR status to the ER/HER2 combined subtypes did not improve the accuracy of the model in predicting the clustering outcome (AIC = 65.881; (Likelihood ratio test; *P* = 1.000). Given these results and since we observed that all 32 different preclinical models cluster on terminal branches, we argue that the adopted normalization strategy was effective in removing batch-specific expression variation, that relevant gene expression themes are preserved, and that replicate gene expression profiles (GEPs) can be reliably averaged.

Averaged GEPs were then used to classify the IBC preclinical models according to their differentiation status using the differentiation predictor model (DPM), the luminal/basal/mesenchymal classification (LBM) system, and PAM50 subtypes. Results are shown in Table 1 and demonstrated that these cell lines all adhered to the basal-like subtype. The majority of the IBC cell lines exhibited a luminal progenitor phenotype (i.e., 7/10) and with respect to the PAM50 classifications, the ER-negative subtypes predominated (i.e., 9/10 basal-like, HER2-enriched or normal like). Notably, all classification distributions, except for the DPM classification (*P* = 0.072) and the HER2 status (*P* = 0.222), are significantly different compared to those obtained in nIBC preclinical models (Table 1).

**Table 1:** Molecular characteristics of IBC models.

IBC model	ER	PR	HER2	ER/HER2	LBM classification	DPM classification	PAM50 subtype
FCIBC01	NEG	NEG	NEG	TN	Basal	Progenitor	Normal
FCIBC02	NEG	NEG	NEG	TN	Basal	Progenitor	Basal
KPL4	NEG	NEG	POS	HER2	Basal	Progenitor	Her2
MaryX	NEG	NEG	NEG	TN	Basal	Progenitor	Basal
MDAIBC3	NEG	NEG	POS	HER2	Basal	Mature	LumA
SUM149	NEG	NEG	NEG	TN	Basal	Progenitor	Basal
SUM190	NEG	NEG	POS	HER2	Basal	Mature	Her2
TJIBC04	NEG	NEG	NEG	TN	Basal	Progenitor	Basal
TJIBC09	NEG	NEG	POS	HER2	Basal	Mature	Her2
UAIBC01	NEG	NEG	POS	HER2	Basal	Progenitor	Her2
*P* value (compared to nIBC)	0.011	0.013	0.222	0.001	0.001	0.072	0.006

Then, in order to investigate if the IBC preclinical models recapitulate biological features typical of IBC in clinical samples, we applied the transcriptomic classifier^9^ consisting of 79 genes with an IBC-specific expression profile on all 32 preclinical models. An elastic net generalized linear model achieved an accuracy of 82% on an independent series (Supplementary Fig. 1). When applied to the series of averaged GEPs of IBC and nIBC preclinical models, an accuracy of 78% was obtained, with a sensitivity and specificity of respectively 100% and 68%. The latter indicated a high rate of false positive predictions amongst the nIBC models (McNemar test; *P* = 0.023), particularly when compared to the patient samples data, where a specificity level of 86% was observed. By consequence, also the positive predictive value was low (i.e., 59%). Another notable observation relates to the fact that when the model was applied onto the replicate GEPs, low posterior probability scores (i.e., close to 0.5) were repeatedly observed for some IBC preclinical models (i.e., SUM149, SUM190, and MDA-IBC-3). All data are shown in Fig. 1b.

To further assess the representativity of the cell lines as models for IBC, the GEPs of UA-IBC-01 and the primary tumor sample it was derived from were directly compared. Both the model and the tumor sample were classified as non-luminal, HER2-enriched according to the PAM50 molecular subtypes despite the use of estrogen pellets during the generation of the UA-IBC-01 PDX-derived cell lines. Gene-wise comparison revealed that both GEPs are strongly correlated (*R*_s_ = 0.740; *P* < 0.001; Supplementary Fig. 2). Out of 12,384 genes expressed above background in both samples, 295 genes were considered overexpressed in the UA-IBC-01 cell line based on expression differences superior to the 97.5th percentile of all gene-wise comparisons. These genes were enriched for hallmark gene sets related to cell proliferation (i.e., E2F target genes: *P* < 0.001; and G2M checkpoint genes: *P* < 0.001). Based on expression differences inferior to the 2.5th percentile of all gene-wise comparisons, 254 genes were considered overexpressed in the primary tumor sample, and these were enriched for gene sets related to immune response programs (i.e., IFNɣ signaling: *P* = 0.007; and TNFɑ signaling: *P* = 0.003) and epithelial-to-mesenchymal transition (*P* < 0.001), which is consistent with the expected enrichment of stroma and immune cells in the primary tumor sample. Interestingly, hallmarks related to hormone receptor signaling (i.e., early estrogen response genes: *P* = 0.012; late estrogen response genes: *P* = 0.002; and androgen response genes: *P* = 0.003) are also enriched amongst genes overexpressed in the primary tumor sample.

### Differential expression and co-expression network analysis

**Fig. 2:**
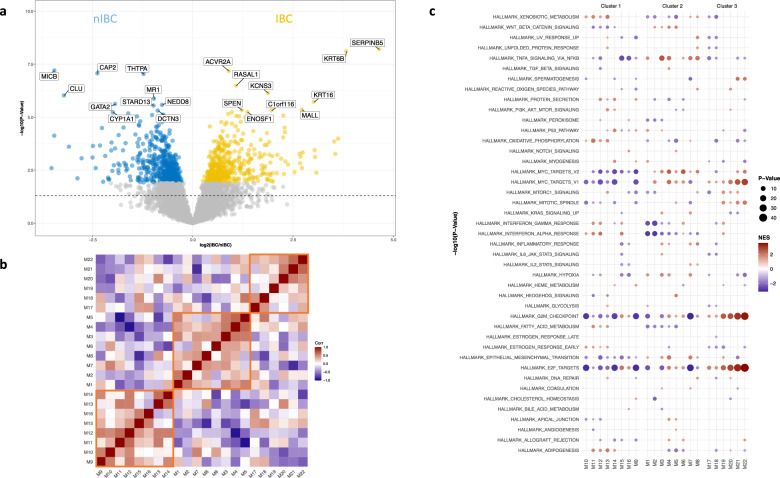
Identification of molecular differences between IBC and nIBC preclinical models. **a** Volcano plot representing gene expression differences between IBC and nIBC preclinical models. The X-axis indicates the log2-transformed gene expression fold change in IBC relative to nIBC. The Y-axis represents the −log10-transformed *p* value. The horizontal dashed line represents a nominal *P* value threshold of 5%. Genes color-coded yellow and blue are overexpressed in IBC and nIBC respectively at a false discovery adjusted *p* value of 10%. The top 10 overexpressed genes in IBC and nIBC cell lines are labeled. **b** Heatmap representing the correlation structure of 22 co-expression modules identified using WGCNA. Pearson correlation coefficients resulting from pairwise comparisons of the eigengenes of the different co-expression modules are coded according to a blue-red color scheme reflecting correlation coefficients ranging from −1 to 1. Row and columns are labeled with the names of the co-expression modules and are ordered according to an unsupervised hierarchical cluster analysis. The three co-expression cluster groups are indicated in orange squares. **c** Dot plot representing the result of a gene set enrichment analysis (GSEA) obtained by comparing the gene-module membership scores for each co-expression cluster to the hallmark gene sets. The co-expression modules are listed along the X-axis and a different facet is created for each co-expression cluster. Enriched hallmarks per modules are indicated using a dot, the color and size of which vary with respectively the normalized enrichment score (i.e., blue = low; red = high) and the −log10-transformed *p* value (i.e., small = less significant; large = more significant) that result from the GSEA.

To identify molecular differences between IBC and nIBC preclinical models, two strategies were applied. First, IBC and nIBC cell lines were compared using generalized linear models to identify differentially expressed genes (DEGs). Hence, 931 DEGs were revealed of which 437 (47%) and 494 (53%) were respectively up- and downregulated in IBC at a false discovery rate of 10%. Results are shown in volcano plot format in Fig. 2a. Differential gene expression statistics are provided in Supplementary Data 1. The resulting fold change vector was then used to perform GSEA for the hallmark gene sets. Results are shown in Supplementary Table 1 and reveal that DEGs overexpressed in IBC cell lines were enriched for gene sets related to IL2/STAT5-, KRAS-, or TP53-signaling and MYC target genes, whereas EMT-related genes were enriched amongst downregulated DEGs.

In a second strategy to characterize the IBC preclinical models, weighted gene co-expression network analysis (WGCNA) was applied onto the averaged GEPs. Using data for all available genes, 22 distinct gene co-expression modules were identified with sizes ranging from 104 to 871 genes. Details regarding the network construction and module detection are shown in Supplementary Fig. 3 and different co-expression module statistics are summarized in Table 2. The correlation structure of the 22 co-expression modules was investigated and revealed the existence of three co-expression clusters (Fig. 2b). Gene set enrichment analysis (GSEA) of the gene-module memberships (GMM) scores (Supplementary Data 2) revealed distinct hallmark enrichment patterns for each of these co-expression clusters (Fig. 2c), suggesting they reflect different biological themes.

**Table 2:** Characteristics of co-expression modules.

Module	Cluster	Size	Hallmark	Preservation Z-Score	Enrichment OR	Enrichment P	CS-MYC	CS-RELA	CS-E2F3
M1	C2	240	HALLMARK_TNFA_SIGNALING_VIA_NFKB	0.585	1.861	0.005	1.660	−85.880	96.080
M2	C2	218	HALLMARK_MYC_TARGETS_V1	0.999	1.061	0.457	−97.950	74.690	−1.980
M3	C2	247	HALLMARK_TNFA_SIGNALING_VIA_NFKB	1.302	1.699	0.015	0.000	−85.490	85.200
M4	C2	786	HALLMARK_MYC_TARGETS_V2	14.426	1.238	0.087	−99.920	−48.210	−60.190
M5	C2	301	HALLMARK_HEDGEHOG_SIGNALING	0.789	2.055	0.000	−5.280	−53.350	15.470
M6	C2	261	HALLMARK_MYC_TARGETS_V2	2.265	1.227	0.234	−93.060	−68.860	−4.220
M7	C2	265	HALLMARK_TNFA_SIGNALING_VIA_NFKB	0.499	1.201	0.260	27.110	−97.240	97.920
M8	C2	871	HALLMARK_TNFA_SIGNALING_VIA_NFKB	5.296	0.577	0.999	−91.210	−52.110	95.200
M9	C1	300	HALLMARK_PROTEIN_SECRETION	9.715	1.281	0.166	99.950	−49.330	−23.640
M10	C1	106	HALLMARK_INTERFERON_ALPHA_RESPONSE	1.786	0.935	0.623	63.750	67.590	2.580
M11	C1	584	HALLMARK_OXIDATIVE_PHOSPHORYLATION	2.656	0.441	1.000	1.660	65.330	6.430
M12	C1	558	HALLMARK_INTERFERON_ALPHA_RESPONSE	20.433	0.771	0.919	99.890	42.620	68.760
M13	C1	199	HALLMARK_XENOBIOTIC_METABOLISM	7.593	0.393	0.994	40.460	65.830	57.160
M14	C1	260	HALLMARK_P53_PATHWAY	6.725	1.159	0.312	99.820	−45.060	1.870
M15	C1	680	HALLMARK_E2F_TARGETS	4.575	0.619	0.995	16.280	95.900	−99.870
M16	C1	141	HALLMARK_E2F_TARGETS	0.537	1.882	0.023	0.000	96.340	5.800
M17	C3	104	HALLMARK_E2F_TARGETS	0.255	0.944	0.613	−12.390	96.070	−16.430
M18	C3	238	HALLMARK_E2F_TARGETS	5.279	2.363	0.000	31.780	−15.840	−77.750
M19	C3	157	HALLMARK_G2M_CHECKPOINT	1.118	0.502	0.967	0.000	71.370	−92.310
M20	C3	124	HALLMARK_E2F_TARGETS	3.928	0.644	0.881	−34.090	92.410	−6.250
M21	C3	148	HALLMARK_E2F_TARGETS	0.541	1.121	0.413	−97.320	78.410	−94.160
M22	C3	394	HALLMARK_E2F_TARGETS	15.215	0.728	0.925	−99.580	95.660	−91.000

*Module* Gene co-expression module, *Cluster* cluster to which the co-expression module belongs, *Size* the number of genes in the module, *Hallmark* top enriched hallmark in the module, *Preservation Z-score* the z-score that defined how significantly a module is conserved in nIBC with values below 2, between 2 and 10 and above 10 indicating respectively poor, moderate, and good preservation, *Enrichment OR* odds ratio for enrichment of genes differentially expressed between IBC and nIBC cell lines in the module, *Enrichment P* *p* value corresponding to the Enrichment OR, *CS-MYC* connectivity score between transcriptional profile of MYC knockdown and the module specific expression profile, *CS-RELA* connectivity score between transcriptional profile of RELA knockdown and the module specific expression profile, *CS-E2F3* connectivity score between transcriptional profile of E2F3 knockdown and the module specific expression profile.

**Fig. 3:**
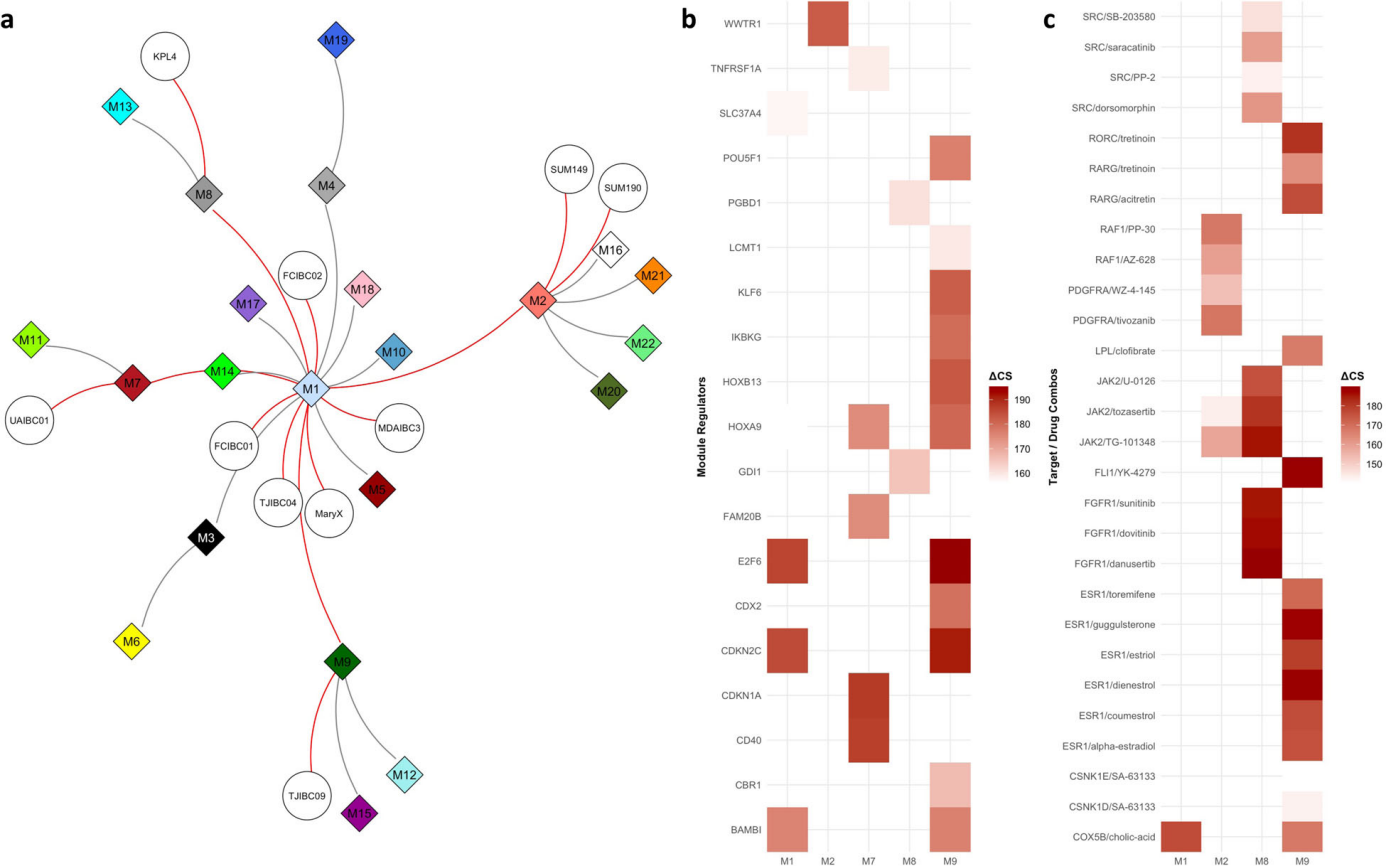
Identification of co-expression cluster regulators and antagonizing chemical compounds. **a** Network diagram showing the minimal set of edges that connect all co-expression modules and cell lines and that were identified using a minimal spanning tree analysis performed on the binary adjacency matrix representing the full set of interactions between all modules and all cell lines. Co-expression modules and cell lines are indicated respectively as diamonds labeled by module number (i.e., M1 to M22) and circles labeled by cell line name. The edges connecting all cell lines through a minimal set of co-expression modules are indicated in red. **b** Results identifying upstream regulators (Y-axis) for each of the five co-expression modules that connect all IBC cell lines (X-axis) are shown in heatmap format. At the intersection between rows and columns, cells are color-coded as shown in the legend only when the difference between the co-expression module specific connectivity Scores (CSs) for overexpression and knockdown of the respective genes exceeds 150 (i.e., at least 75 upon overexpression and at most -75 upon knockdown). **c** Results identifying target/drug combinations (Y-axis) for each of the five co-expression modules that connect all IBC cell lines (X-axis) are shown in heatmap format. At the intersection between rows and columns, cells are color-coded as shown in the legend only when the difference between the co-expression module specific CSs for overexpression of the drug target and drug treatment exceeds 150 (i.e., at least 75 upon overexpression of the target and at most −75 upon treatment).

The extent to which each of the co-expression modules is preserved in the gene expression series of the nIBC preclinical models was investigated (Table 2). The highest preservation score was obtained for the module containing *ERBB2* (i.e., M12), most likely reflecting the presence of ERBB2+ cell lines in both IBC and nIBC series. The module containing *ESR1* (i.e., M10) was poorly conserved (i.e., preservation score inferior to 2), probably due to the fact that all IBC preclinical models are ER-negative and thus the ER-related expression patterns in IBC preclinical models are weaker than in nIBC. Overall, the IBC co-expression modules contained in the 2nd co-expression cluster, which were associated with amongst others MYC, NFκB, and Hedgehog signaling (Table 2), were most weakly conserved in nIBC cell lines (i.e., average preservation score per cluster group: C_1_ = 6.753; C_2_ = 3.270; and C_3_ = 4.390), suggesting that these gene clusters reflect biological themes that are more intrinsic to IBC. This is corroborated by the enrichment of genes overexpressed in IBC cell lines (*vide supra*) in 4/8 of the co-expression modules in the 2nd co-expression cluster (Table 2). Finally, based on the correlation structure of the 22 co-expression modules (Fig. 2b) and their cell line-specific expression levels, a network-based prioritization of the co-expression modules was performed. A minimal set of five co-expression modules connecting all IBC cell lines was identified (i.e., M1, M2, M7, M8, and M9), all but one belonging to the 2nd co-expression cluster (Fig. 3a).

### Identification of co-expression cluster regulators and antagonizing chemical compounds

Based on the co-expression modules analysis, we then aimed to identify potential modulators of IBC biology as well as potential drug/target combinations for therapy using the CMAP data set. Connectivity Scores (CSs) are provided in Supplementary Data 3 for all comparisons. As a proof-of-concept, we focused on the CSs of RELA and MYC and demonstrate that knockdown of these transcription factors induced a gene expression profile that was opposite to the characteristic expression profile of the gene co-expression modules enriched for genes involved in NFκB and MYC signaling respectively (Table 2 and Supplementary Fig. 4). In addition, we also evaluated all E2F transcription factors (i.e., E2F1 to E2F9) and revealed that particularly the CSs associated with E2F3 knockdown were reduced in those expression modules enriched for E2F target genes. For other E2F transcription factors, no clear association was observed.

We then focused on the set of five co-expression modules (M1, M2, M7, M8, and M9) linking all 10 IBC cell lines to reveal potential regulators and drug/target combinations. According to published literature, several of the identified regulator genes (Fig. 3b) were related to MYC activity (i.e., *BAMBI, CD40, CDKN1A, CDKN2C, CDX2, E2F6, HOXA9, HOXB13, KLF6, POU5F1*, and *WWTR1/TAZ*), often in conjunction with WNT signaling, TGFβ signaling or stem cell biology. In addition, 31 potentially effective drug/target combinations were identified involving 13 distinct targets and 26 different drugs (Fig. 3c). Unfortunately, no single target/drug combination was predicted to be effective in all five co-expression modules and no target/drug combination meeting our criteria was identified for M7. Remarkably, for M9 that connects to TJ-IBC-09, our data suggested sensitivity to anti-hormonal drugs.

### MYC expression and transcriptional activity in IBC patient samples

**Fig. 4:**
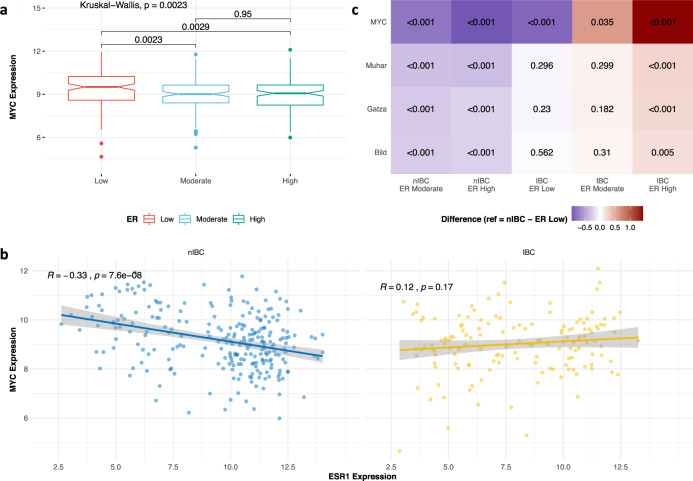
MYC expression and transcriptional activity in patient samples in function of ESR1. **a** MYC expression levels (Y-axis) in a data set of 146 IBC and 252 nIBC tissue samples classified according to the ER status, calculated by stratifying ESR1 mRNA levels into low, moderate, and high categories. Data are represented in notched boxplot format and color-coding according to the legend shown underneath the plot. *P* values resulting from the pairwise comparison of the MYC expression distributions between the different tumor sample categories are indicated. **b** Scatter plot comparing ESR1 and MYC expression, represented in the X- and Y-axis respectively, in nIBC (left) and IBC (right) patient series. For each series, a trend line is plotted and the resulting Spearman correlation coefficients are shown in the top left corner. **c** Heatmap representing the result of a generalized linear regression analysis evaluating MYC expression levels (i.e., top row) and MYC transcriptional activation calculated using the gene sets published by Muhar et al., Gatza et al. and Bild et al. (i.e., bottom 3 rows) in function of the tumor phenotype (i.e., IBC vs. nIBC) and interactions thereof with different strata of ER expression. For each of the resulting categories, shown along the X-axis (i.e., nIBC—ER Moderate, nIBC—ER High, IBC—ER Low, IBC—ER Moderate, and IBC—ER High), regression coefficients representing the change in MYC expression or MYC transcriptional activity in that category relative to nIBC samples with low ER expression, are color-coded as shown in the legend. The heatmap for example shows that MYC expression (i.e., top row) in nIBC samples with high ER levels (i.e., 2nd column) decreases significantly (i.e., blue color) as compared to nIBC samples with low ER expression, whereas relative to the same category the MYC expression in IBC samples with high ER levels increases significantly. *P* values evaluating the significance of the changes are indicated in each cell.

Our results in the preclinical models described above suggested that MYC could be an important driver of IBC biology. To corroborate these data, MYC-related molecular changes were evaluated in our series of 146 and 252 expression profiles from IBC and nIBC tissue samples^9^. As shown in Fig. 4a, MYC expression was dependent on the ER status defined by stratifying *ESR1* mRNA levels into low, moderate, and high expression categories (*P* < 0.003). When comparing IBC to nIBC, no significant difference in MYC expression was observed (*P* = 0.209). However, when stratifying by ER status, MYC expression was significantly different between IBC and nIBC (*P* = 0.049). Correlation analysis (Fig. 4b) revealed a significant inverse relation between *ESR1* and *MYC* expression in nIBC (*R*_s_ = −0.331; *P* < 0.001). Similar correlations were also noted in the TCGA and METABRIC series that primarily consist of nIBC tissue samples (TCGA: *R*_s_ = −0.250; *P* < 0.001; METABRIC: *R* = −0.222; *P* < 0.001; data not shown). In IBC however, a different correlation pattern was observed (*R*_s_ = 0.115; *P* = 0.166), suggesting different interactions between ER and MYC depending on the tumor phenotype. A generalized linear model testing for such interactions, demonstrated that *MYC* expression in nIBC indeed decreased with increasing *ESR1* levels (i.e., decrease with 0.754 and 0.931 expression units in respectively the ER moderate and high categories relative to the ER low category; all *P*s < 0.001). Results are shown in Fig. 4c, in which the first two columns represent the ER moderate and ER high categories in nIBC. In IBC samples with low *ESR1* levels, *MYC* expression was 0.832 units lower as compared to nIBC samples with similar *ESR1* mRNA levels (*P* < 0.001; third column in Fig. 4c) and *MYC* expression increased by 0.599 and 1.414 expression units in respectively the ER moderate and high categories (*P* = 0.035 and *P* < 0.001 respectively; fourth and fifth column in Fig. 4c). To evaluate differences in MYC transcriptional activity between IBC and nIBC samples, a similar analysis was performed using activation scores calculated using GSVA based on three different published MYC activation signatures^32–34^. Hence, we noticed that differences in MYC transcriptional activity followed the same trends as those described for MYC expression, with the exception that MYC transcriptional activity was not different between IBC and nIBC samples with low ER expression (Fig. 4c).

To evaluate potential confounding effects in the reported observations, MYC expression and transcriptional activation were first compared between samples stratified by tumor stage and the PAM50 subtypes. Significant MYC expression and activation differences according to the strata of both classification systems were observed (Supplementary Figs. 5, 6). Incorporating tumor stage or the PAM50 subtypes separately as blocking variables into each of the generalized linear models described above, revealed that tumor stage did not confound the observed interaction differences of ER and MYC between IBC and nIBC. The PAM50 subtypes on the other hand did account for the overall effect of ER on MYC expression or activation, but the IBC-specific associations between ER and MYC remain significant in 3/4 comparisons. Results, comparing the original with the blocked regression models for each MYC-related feature, are shown in Supplementary Table 2.

### Expression analysis of proximal MYC network members

**Fig. 5:**
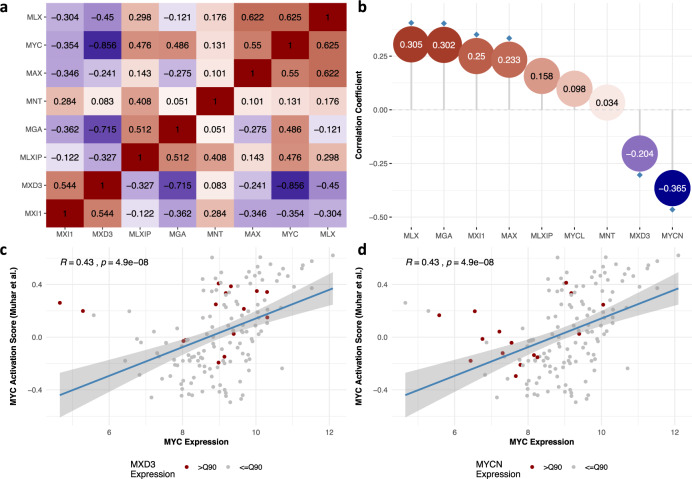
Expression analysis of proximal MYC network members. **a** Heatmap showing the correlation structure of the proximal MYC network members in IBC cell lines. The proximal MYC network members are indicated along both X- and Y-axes and ordered similarly based on the output of a Ward clustering analysis. Correlations are coded according to a blue-red coloring scheme representing negative to positive correlation coefficients respectively. Correlation values are provided in the corresponding cells. **b** MYC correlation analysis for the proximal MYC network members in a series of 146 IBC patient samples. The proximal MYC network members and strength of the correlation between their expression and MYC mRNA levels are shown along the X- and Y-axis respectively. Each correlation coefficient is indicated by a dot, coded according to a blue-red coloring scheme representing negative to positive correlation coefficients respectively and correlation values are provided inside. Significant values are indicated with a blue diamond. **c**, **d** Scatter plots comparing MYC expression (X-axis) and MYC transcriptional activity (Y-axis) calculated according to the gene set published by Muhar et al. in a series of 146 IBC samples. Each sample is represented by a point, which is colored red if the corresponding sample is characterized by MXD3 (**c**) or NMYC (**d**) expression values exceeding the 90th percentile. A regression line depicting the linear relationship between MYC expression and transcriptional activity is indicated and results of the Spearman correlation analysis are provided in the top left corner.

To provide additional context to the role of MYC in IBC biology, expression levels of genes belonging to the Proximal MYC Network (PMN) and that modulate MYC target gene binding and expression^35^, were investigated. With the exception of MYC itself, mRNA levels for only seven members were available in the series of 10 IBC cell lines and the correlation plot is shown in Fig. 5a. This reveals that expression levels MAX (i.e., the primary interaction partner of MYC; *P* = 0.099) and MLX (i.e., MAX-like protein, another dimerization partner of MYC; *P* = 0.053) were positively correlated with MYC, whereas a negative correlation was reported for MXD3 (i.e., MYC competitive MAX dimerization partner; *P* = 0.001). These relationships were recapitulated in a series of 146 samples from patients with IBC (i.e., correlation *P* values for MAX: *P* = 0.005, MLX: *P* < 0.001 and MXD3: *P* = 0.027; Fig. 5b). In addition, an inverse relation between MYC and MYCN (i.e., another member of the family of MYC transcription factors) was noted (*P* = 0.016). In general though, the correlation strengths among the PMN members observed in tissue samples were weaker as compared to those obtained in cell lines, possibly owing to confounding effects of ER expression and stromal admixture.

Finally, in the series of IBC patient samples, we demonstrated that MYC transcriptional activation, calculated based on the target gene set published by Muhar et al.^32^ was positively associated to MYC (*P* < 0.001), NMYC (*P* = 0.017), MLX (*P* = 0.045) and MXD3 (*P* < 0.001) and negatively to MAX (*P* < 0.001). Identical results were obtained for the MYC transcriptional activation scores calculated using the two remaining gene sets (data not shown). Figure 5C, D show the strong linear relationship between MYC expression and MYC transcriptional activation but also identify a set of outlier samples in which MYC transcriptional activation was associated with elevated mRNA levels of either *MXD3* of *NMYC* rather than *MYC* itself.

## Discussion

The primary goal of the current study was to gain insight into the molecular characteristics of ten IBC cell lines and to determine to what extent these preclinical models reflect genuine IBC biology. Therefore, gene expression data of IBC preclinical models were integrated with publicly available expression data of nIBC preclinical models. Data set-dependent bias was efficiently removed by normalization as shown by the clustering of replicate samples on terminal branches and by the fact that biologically relevant expression themes, such as those related to the ER and HER2 status of the cell lines, were not compromised. To further limit the effect of gene expression fluctuations associated with passage number, different culture conditions or other stochastic variables, the molecular profile of each preclinical model in this study was defined as the average of several replicates, except for the UA-IBC-01 cell line for which only one expression profile was available.

Based on the resulting data set, we observed that all IBC cell lines adhere to the basal-like subtype and 7/10 had a luminal progenitor phenotype. This agrees with the fact that all IBC cell lines are ER-negative and demonstrates that the degree of molecular heterogeneity in the present series of IBC preclinical models is restricted as compared to the nIBC cell lines in which all subtypes and differentiation states are represented. Similar conclusions could be drawn when evaluating the distribution of the PAM50 subtypes, with 8/10 IBC cell lines being either basal-like or HER2-enriched. These results can be explained by the dominance of the basal-like and HER2-enriched subtypes in IBC tissue samples^9,36^. However, they also clearly reveal the paucity in ER+, luminal-type preclinical IBC models despite the fact these subtypes account for roughly 30–40% of the patient samples^14,37^. Another intriguing observation is the absence of the mesenchymal subtype in the panel of IBC cell lines, which is unexpected due to the metastatic potential often associated with this subset of breast cancer cells, but agrees with the reported overexpression of E-Cadherin in IBC cells and thus their presumed epithelial phenotype^38^.

Next, we aimed to evaluate to what extend the current series of IBC preclinical models is representative for IBC biology. Therefore, we applied a classification model based on 79 genes with IBC-specific expression patterns^9^ onto our preclinical model series. Overall, the prediction accuracy was acceptable (i.e., 78%), but positive predictive value was limited (i.e., 59%) indicating that the model was not reliable in correctly predicting the IBC status of the models or that the models do not fully represent the clinical disease but only limited aspects of it. In an additional analysis, we observed that overexpression patterns in nIBC were maintained between cell lines and tissue samples, but not those from IBC cell lines (data not shown). This result suggests that separation of IBC and nIBC cell lines based on the transcriptomic classifier is mainly driven by the nIBC marker genes. A possible explanation for the lack of predictive power associated with the IBC marker genes in this analysis relates to differences between IBC and nIBC associated with the tumor (immune) microenvironment^13–15,39^ that are not recapitulated in the data set of the preclinical models. Indeed, 67% of the IBC marker genes that are part of the IBC-specific transcriptomic signature demonstrate elevated expression in the profiles of immune and endothelial cells of the Human Tissue Compendium relative to those of epithelial cells (data not shown). This conclusion is further corroborated by a direct comparison of the GEPs of the UA-IBC-01 cell line and the primary tumor sample it is obtained from. Although expression differences are limited and cancer cell intrinsic expression themes appear to be preserved in the preclinical model, enrichment of gene sets associated with immune response programs in the primary tumor sample were noted. Together, these data again underscore the importance of the TME in IBC biology, but they do not preclude the utility of the preclinical models to uncover IBC cell intrinsic features and numerous efforts are underway to incorporate immune features into programs for the development of IBC preclinical models. With respect to the latter, the use of estrogen supplementation should be carefully considered, as modest differences in gene expression of estrogen response genes were noted between UA-IBC-01 and its corresponding primary tissue sample. However, since gene expression changes are not in line with the expected results, this observation requires further investigation.

To further delineate the defining principles of IBC biology, two distinct analysis strategies were undertaken. First, DEGs between IBC and nIBC cell lines were identified. Second, WGCNA was applied to detect modules of co-expressed genes in the series of IBC cell lines only. In both analyses, confounding variables such as ER status and the molecular subtypes were not taken into account for two reasons. First, our data set was too small to perform multivariate analyses. Second, since all the IBC preclinical models are ER-negative and of the basal-like subtype, it is impossible to reliably differentiate between gene expression patterns introduced by the tumor phenotype on the one hand and other sources of latent variation on the other hand. It should be noted that the inability to account for covariates represents a debilitating factor in our analysis. Regardless, both strategies pointed at important roles for cell proliferation (e.g., hallmarks E2F target genes and G2M checkpoint), MYC transcriptional activity and inflammatory response programs (e.g., hallmarks IL2/STAT5 and TNFɑ/NFκB) in the biology of IBC. Furthermore, the WGCNA approach revealed that several of these processes are jointly regulated. For example, MYC and NFκB target gene expression were the dominant themes in the second co-expression cluster, which suggests an intricate relationship between both transcription factors in shaping IBC biology. NFκB activation in IBC has been reported previously^29,40–42^, and MYC and NFκB have been shown to cooperate in breast cancer development and progression^43–46^, amongst others by modulating stem cell behavior. By comparing gene co-expression clusters to the L1000 profiles of the CMAP database, we also revealed sets of potential regulators and target/drug combinations. Unfortunately, no single drug with predicted efficacy across all IBC cell lines was identified. This may reflect inherent heterogeneity in the signal transduction networks of the individual IBC cells, limited specificity of the CMAP profiles that are generated using a broad collection of cancer cell lines and thus are not reflective of breast cancer specific transcriptional responses, or the failure to identify robust and highly specific IBC co-expression modules due to the limited size and relative homogeneity (i.e., all ER-negative and basal-like samples) of the series of IBC preclinical models. Nevertheless, our results constitute a good starting point to evaluate novel treatment strategies in preclinical IBC research.

The fact that MYC target genes were overexpressed in IBC cell lines and were associated with specific co-expression modules suggests that MYC transcriptional activity is an important characteristic of IBC biology at least in preclinical models. This is corroborated by earlier work of Zhang et al. who demonstrated that MYC is a central hub in the signal transduction networks of SUM149 and SUM190^47^. In addition, we recently showed that MYC mediates the specific response of IBC cells to TGFβ1 treatment^10^. To confirm these observations, MYC expression and transcriptional activation were explored using GEPs from IBC and nIBC tissue biopsies. Our data demonstrate that the levels of *MYC* expression and transcriptional activation in relation to *ESR1* expression exhibit opposite patterns in IBC and nIBC with an ER-dependent decrease and increase in mRNA levels of both *MYC* and MYC target genes in nIBC and IBC respectively. Particularly the positive association between ER and MYC expression or transcriptional activation in IBC is notable, as it remains significant even when accounting for tumor stage and the PAM50 subtypes and thus cannot be attributed solely by the enrichment of the Luminal B phenotype amongst ER+ IBC tissue biopsies. This observation is in line with earlier results showing that MYC is a common denominator of biological processes active in ER-positive IBC^48^. The induction of MYC activity in ER-positive IBC may provide an explanation for the hormone therapy resistance phenotype often associated with IBC^49^. Miller et al. illustrated that a gene signature of breast cancer cells with acquired hormone independence and predictive of resistance to hormonal therapy reflects MYC pathway activation^50^. Importantly, these data re-emphasize the need for ER+ luminal-type preclinical models of IBC. It stands to reason that inclusion of such models in our present analysis would further amplify the here reported MYC-related differences.

Recently, we reported that MYC was frequently affected by genomic alterations in a series of 101 IBC tissue biopsies^12^ and Faldoni et al. reported frequent gains covering the MYC gene in IBC, with a concomitant MYC protein overexpression in IBC patient samples^51^. We performed a meta-analysis of published data^12,52–56^ and demonstrate that the frequency of MYC genomic alterations in primary IBC is 23% (95% CI: 13–33%) vs. 30% (95% CI: 24–37%) in a subtype matched nIBC series consisting of METABRIC and TCGA samples respectively. This reveals that MYC genomic alterations are not specifically enriched in IBC. Therefore, we hypothesize that other mechanisms of MYC activation in IBC are operational and the present data contribute to earlier observations linking MYC in IBC to signaling pathways involved in developmental biology^12,57^. Here, we show that some co-expression modules associated with MYC target gene expression are additionally related to WNT or Hedgehog signaling, and that many of the upstream regulators for these co-expression clusters are also involved in these pathways (i.e., BAMBI, E2F6, HOXB13, and WWTR1/TAZ) or in plain stem cell biology (i.e., CDX2, HOXA9, KLF6, and POU5F1). Interestingly, a SNP (rs6983267) located at 8q24 close to the MYC locus, is known to exhibit enhanced binding properties for the WNT effector TCF4 and can be directed to the MYC locus through chromatin loops allowing for WNT/TCF4-dependent MYC expression. In addition, the rs6983267 genotype is associated with metastatic risk in IBC but not in nIBC, suggesting that MYC is also involved in cancer cell dissemination in IBC^58^. In line with this, MYC expression levels are predictive of reduced distant metastasis-free survival in patients with ER-positive IBC^48^ and have shown to be associated with metastasis in ER-positive metastatic breast cancer^59,60^. Apart from stem cell signaling pathways, also signals from the TME could be involved in modulating MYC signaling. In this context, MYC has been shown to be a target gene of the NFκB transcription factor RELA^43,61^ that orchestrates cellular responses to pro-inflammatory cues. Finally, we want to draw attention to the fact that in a small subset of IBC samples, the activation of the MYC pathway was apparently associated with NMYC and MXD3, which have been shown to jointly regulate cell proliferation in cerebellar granule neuron precursors, downstream of Hedgehog signaling^62^. This indicates that MYC biology in IBC is complex and involves different PMN members.

In conclusion, in this study we demonstrate that the currently available preclinical models of IBC recapitulate to some extent the molecular features of IBC cells in patient samples, and thus constitute valuable research tools. However, it should be noted that the present panel of IBC models do not fully recapitulate the molecular heterogeneity seen in patient samples. Particularly, the lack of hormone sensitive, luminal-type preclinical models for IBC is worrisome, since data indicate that ER may contribute to IBC biology in a specific manner as shown by the ER-dependency of MYC expression and transcriptional activity in patient samples. By consequence, the lack of ER expressing IBC cell lines represents one of the major limitations associated with the present study, particularly in the comparison of IBC and nIBC cell lines in which the influence ER positivity on differences in gene expression could not be assessed. A second limitation of this study and of the presented series of preclinical models in general relates to the absence of the specific immune contexture, which is now being increasingly accepted as a hallmark of IBC biology. However, it should be noted that signatures of activated immune response pathways prevail in IBC cells as intrinsic properties, possibly reflecting past interactions between IBC cells and an inflamed tumor microenvironment. Finally, the rather limited size of the series of IBC cell lines, which impacts on statistical power, implies that additional and more subtle molecular features of IBC cells may yet be undetermined. We argue that researchers need to be aware of these limitations, allowing their appropriate consideration in the design of preclinical experiments to maximize the translatability of research results into daily patient care.

## Methods

This study was approved by the local review board of the GZA Hospitals and each patient gave written informed consent.

### UAIBC01 PDX model

The UAIBC01 PDX model was generated in collaboration with Oncotest Gmbh (Freiburg, Germany). Briefly, metastatic tumor tissue from a patient with hormone receptor-negative and HER2-amplified IBC was subcutaneously implanted in NOD-SCID mice with estrogen pellets and serially passaged in nude mice. Tissue samples obtained after the fourth passage were processed for molecular analysis. Information on the clinical and pathological characteristics of the patient from which this IBC cell line and the other nine IBC models were derived, can be found in Supplementary Table 3.

### Gene expression data from cell lines

We profiled a series of nine IBC (i.e., SUM149, SUM190, KPL4, Mary-X, MDAIBC03, FCIBC01, FCIBC02, TJIBC04, and TJIBC09) and three nIBC (i.e., MCF7, MDAMB231, and SUM159) preclinical models at least in triplicate using Affymetrix HGU133plus2 GeneChips. Using the same platform, an additional gene expression profile (GEP) of our in-house generated PDX model for IBC (i.e., UAIBC01) was generated and included in the study, yielding a total of 57 GEPs. The SUM149, SUM190, KPL4 and MDAIBC03 cells were a kindly gift from MD Anderson Cancer Center, TX, USA. The FCIBC01, FCIBC02, TJIBC04, and TJIBC09 cells were a kindly from M.C. and the Mary-X model from Dr. Barsky. The nIBC cell lines were purchased from ATCC (Manassas, USA).

To expand the group of nIBC preclinical models, four additional gene expression data sets generated using the Affymetrix HGU133 series were retrieved from public resources (Gene Expression Omnibus: GSE12777, GSE16795, and GSE40464; and ArrayExpress: E-MTAB-7). Expression data for 19 extra nIBC models was avalaible, i.e., BT-20, BT-474, BT-483, BT-549, CAMA1, HCC1937, HS578T, MDAMB134VI, MDAMB175VII, MDAMB361, MDAMB415, MDAMB436, MDAMB453, MDAMB468, SKBR3, T47D, UACC812, ZR751, ZR7530. To reduce technical bias due to interlaboratory variability in these data sets, only cell lines that were profiled at least in triplicate were included. In total, 124 expression profiles of 32 different breast cancer models (i.e., 10 IBC models and 22 nIBC models) from five different data sets were included.

For each individual data set (*N* = 5), expression data were normalized using the Robust Multi-array Averaging algorithm with correction for GC probe content (BioConductor package *gcrma*—v.2.60.0) and probe sets with a fluorescence intensity above log_2_(100) in at least two samples were included. The individual data sets were then merged based on the common probe sets (*N* = 10,961) and batch effects were removed using empirical Bayesian methods (i.e., the combat function implemented in the BioConductor-package *sva*—3.36.0), with protection of cell line-specific variation in gene expression. The resulting data set was then subjected to quantile normalization and probe set redundancy was removed by retaining the probe set with the highest standard deviation *per* gene for a total of 7182 unique genes. As a final step, replicate GEPs were averaged.

### Gene expression data from patient samples

Gene expression data from 146 IBC and 252 nIBC tissue samples have been described earlier^9^. However, in this study, raw GEPs were reprocessed using a similar normalization strategy as described for the preclinical models in order to ensure data comparability. Batch effects due to the inclusion of samples from three distinct sites (i.e., MD Anderson, Institut Paoli-Calmettes and GZA Hospitals Sint-Augustinus) were removed using empirical Bayesian methods. The final processed data set contained 12,769 probes sets for 8086 unique genes. Finally, breast cancer gene expression data from the TCGA (Firehose legacy) and METABRIC series were downloaded from the cBioPortal for cancer genomics (https://www.cbioportal.org) using the R package *cgdsr* (v.1.3.0).

### Unsupervised analysis

UHCA was performed using Manhattan distance as dissimilarity metric and Ward clustering as the dendrogram drawing method. Prior to cluster analysis, data were centered and scaled to unit variance. The optimal number of clusters, ranging from 2 to 10, was determined by evaluating cluster separability based on 30 distinct indices (BioConductor package *NbClust*—v.3.0). The clustering scheme that was supported by the majority of these indices was selected.

### Classification

Preclinical models and patient samples were classified according to the PAM50 molecular subtypes^63^ using the BioConductor package *genefu* (v.2.20.0). Furthermore, the preclinical models were classified according to the ER status, the PR status, the HER2 status^64^, the luminal/basal/mesenchymal classification (LBM) system for breast cancer cell lines^65^, the DPM^66^, and the IBC-specific classification model composed of 79 probe sets^9^. For the PR status, nIBC cell lines reported to be PR negative by Dai et al. were considered as PR negative whereas nIBC cell lines with weak or strong PR expression were classified as PR positive^64^. In addition, IBC and nIBC patient samples were stratified into ER low, moderate and high expression groups based on the 33rd and 66th quantiles of the *ESR1* mRNA levels.

To perform the classification according to the signature of 79 IBC-specific probe sets, a model based on elastic net generalized linear regression was optimized on GEPs of the tumor samples using the R package *caret* (v.6.0-86). This data set was split into a training and validation set according to a 3/1 split ratio. Prior to model construction, the data were centered and scaled to unit variance. Model construction was performed using repeated tenfold cross-validation against a tuning grid of alpha values ranging from 0 to 1 with 0.1 increments and lambda values ranging from 0.001 to 0.1 with 0.001 increments. The optimal model was selected using ROC statistics and was then applied onto the validation set of tumor samples to define the model accuracy and onto the data set of preclinical models to record posterior probabilities for each breast cancer model. For the latter analysis, the non-averaged GEPs (*N* = 124) were used in order to be able to evaluate the cell line-specific variation of the classification scores and the final call was generated based on the median posterior probability across replicates.

### Differential expression analysis

Identification of DEGs was performed using the BioConductor package *limma* (v.3.44.3). Resulting *p* values were corrected for false discovery using the Benjamini and Hochberg procedure and false discovery rate (FDR)-corrected *p* values inferior to 10% were considered significant.

To identify genes presumably differentially expressed between the UA-IBC-01 preclinical models and to primary tumor sample it was derived from, gene-wise differences in expression between both samples were calculated by subtracting the expression levels measured in the cell line from those measure in the tumor sample. Genes overexpressed in the tumor sample and the cell line were then defined based on respectively the 97.5th and the 2.5th percentile of the global distribution of the gene-wise expression differences.

### Weighted gene co-expression network analysis (WGCNA)

To identify gene co-expression modules in the series of 10 IBC preclinical models, WGCNA was performed using the R package *WGCNA* (v.1.69) and following online instructions (https://horvath.genetics.ucla.edu/). To construct a signed co-expression matrix, pairwise biweight midcorrelation coefficients were calculated between all 7182 genes. The resulting adjacency matrix was transformed into a weighted network by raising the biweight midcorrelation coefficients to a power that was chosen for the resulting network to adhere to scale-free topology. Detection of co-expression modules was performed using UHCA by subjecting the topological overlap dissimilarity matrix of the network to Ward clustering. The resulting dendrogram was analyzed using an adaptive branch pruning algorithm combined with partitioning around medoids to assign genes to co-expression clusters enforcing a minimum size of 100 genes and co-expression clusters with a similar profile were merged. Then, GMM scores were calculated for each gene and each co-expression cluster and represent the Pearson correlation coefficient between their respective expression profiles. The vector of all gene-wise GMM scores per module is considered as the characteristic GEP of that module. The preservation of the IBC co-expression clusters in the series of nIBC preclinical models was investigated using connectivity and density statistics. Finally, network-based prioritization of the co-expression clusters was performed using the R package *igraph* (v.1.2.5). Therefore, based on the expression levels of the co-expression modules in the cell lines, the correlation structure amongst the co-expression modules was determined and dichotomized relative to 0 (i.e., positive and negative correlation coefficients transformed into 1 and 0 respectively). Then, individual cell lines were linked to the co-expression modules by dichotomizing the cell line-specific expression values of the co-expression modules (i.e., positive and negative expression values transformed into 1 and 0 respectively). The resulting binary adjacency matrix, representing both modules and cell lines, was analyzed using a minimal spanning tree algorithm to determine the minimal set of edges that connect all components. The result was visualized using the R package *ggnetwork* (v.0.5.8).

### Systems biology

To translate expression profiles (i.e., vectors of log_2_-transformed fold changes or GMM scores) into biological themes, GSEA was performed for the hallmark gene sets of the molecular signatures database (https://www.gsea-msigdb.org/gsea/msigdb). Overrepresentation analysis (ORA) of DEGs between IBC and nIBC cell lines in each of the co-expression modules was performed using the hypergeometric test. Both analyses were performed using the BioConductor package *fgsea* (v.1.14.0). To assess MYC transcriptional activity in IBC and nIBC tissue samples based on published MYC activation signatures^32–34^, GEPs were subjected to gene set variation analysis using the BioConductor package *GSVA*. Finally, the analysis of the PMN was performed based on genes reported by Schaub et al.^35^.

To identify regulators of IBC biology and potentially effective target/drug combinations for treatment based on the WGCNA results, the GEP of each co-expression module based on 300 marker genes with the highest or lowest GMM scores (i.e., 150 each) was analyzed against 1.3 million L1000 profiles present in the CMAP data set (https://clue.io/cmap). These L1000 profiles catalog the transcriptional responses of human cells to a variety of chemical or genetic (i.e., both knockdown and overexpression) perturbations. The resulting connectivity scores (CSs) reflect the level of agreement between the analyzed GEPs and the L1000 profiles and range between −100 and 100 reflecting incongruent or congruent profiles respectively. Then, for each co-expression module, regulators are defined as genes with a CS of at least 75 upon overexpression and at most −75 upon knockdown and drug/target combinations are defined based on a CS smaller than −75 for the drug and greater than 75 upon overexpression of the drug target.

### Statistics

To compare the distribution of two categorical variables, Fisher Exact tests, Chi-square tests or multinomial regression analyses were performed. To compare the distribution of a continuous variable in the context of one or more categorical variables, Wilcoxon tests, Kruskal–Wallis tests or generalized linear regression analyses were performed. To compare two continuous variables, Spearman correlation or linear regression analyses were performed. Regression models were performed in uni- or multivariate setting where appropriate. Particularly, to analyze MYC-related parameters in the context of ER expression and the tumor phenotype, a nested interaction model was established to estimate the main effect of the tumor phenotype and ER expression in addition to IBC-specific effects of ER expression on MYC expression levels. Comparison of different regression models was performed using the likelihood ratio test. In all cases, two-sided tests were performed and *p* values inferior to 5% were considered significant. Data analysis was done in R (v.4.0.1) and data visualization was done using the R package *ggplot2* (v.3.3.1).

### Reporting summary

Further information on research design is available in the Nature Research Reporting Summary linked to this article.

## Supplementary information

Supplementary Information

Supplementary Data 1

Supplementary Data 2

Supplementary Data 3

Reporting Summary

## Data Availability

The data that support the findings of this study are available from the corresponding author upon reasonable request. The GEPs of the ten IBC preclinical models and three nIBC preclinical models (i.e., MCF7, MDAMB231, and SUM159) can be accessed on ArrayExpress with accession number E-MTAB-11134.

## Code availability

All code generated in part of this publication is available at https://github.com/StevenVanLaere/IBCModels.
