# Supplementary material for: Comparative transcriptional analyses of preclinical models and patient samples reveal MYC and RELA driven expression patterns that define the molecular landscape of IBC
Source: NPJ Breast Cancer. 2022 Jan 18;8:12. doi: 10.1038/s41523-021-00379-6 (PMC8766434; doi:10.1038/s41523-021-00379-6)
Supplement: Supplementary file 1 — Supplementary Information [file 41523_2021_379_MOESM1_ESM.pdf]

SUPPLEMENTARY INFORMATION FOR

Comparative transcriptional analyses of preclinical models and patient samples reveal MYC and RELA driven expression patterns that define the molecular landscape of IBC.

Summary:

SUPPLEMENTARY FIGURES
Supplementary Figure 1: Results of the model optimization and application
Supplementary Figure 2: Genome-wide expression levels measured in the UA-IBC-01 cell line in function of those measured in the corresponding primary tumor sample.
Supplementary Figure 3: Network construction and module detection.
Supplementary Figure 4: Connectivity scores for MYC, RELA, and E2F3 knockdown for each of the co-expression clusters
Supplementary Figure 5: Boxplots showing MYC expression and MYC activation according to published gene signatures in function of tumor stage
Supplementary Figure 6: Boxplots showing MYC expression and MYC activation according to published gene signatures in function of the PAM50 subtypes

SUPPLEMENTARY TABLES
Supplementary Table 1: Gene set enrichment analysis to identify hallmark gene sets
Supplementary Table 2: Regression analysis for each MYC-related feature
Supplementary Table 3: Clinical and pathological information of the patients (n=10) from whom the IBC models were derived.

SUPPLEMENTARY DATA
Supplementary Data 1: Differential gene expression data comparing IBC and nIBC models
Supplementary Data 2: Gene set enrichment analysis (GSEA) of the gene module memberships (GMM) scores.
Supplementary Data 3: Connectivity Scores to identify potential modulators of IBC biology as well as potential drug/target combinations for therapy using the CMAP dataset.

SUPPLEMENTARY FIGURES

A

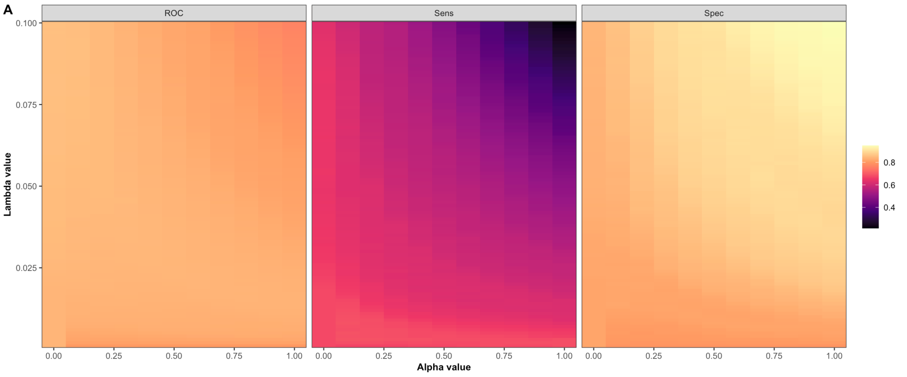

B

Statistic	Tissue	Models
Accuracy	0.818	0.781
Kappa	0.607	0.573
AccuracyLower	0.728	0.600
AccuracyUpper	0.889	0.907
AccuracyNull	0.636	0.688
AccuracyPValue	0.000	0.172
McnemarPValue	1.000	0.023
Sensitivity	0.750	1.000
Specificity	0.857	0.682
Pos Pred Value	0.750	1.000
Neg Pred Value	0.857	0.855

C

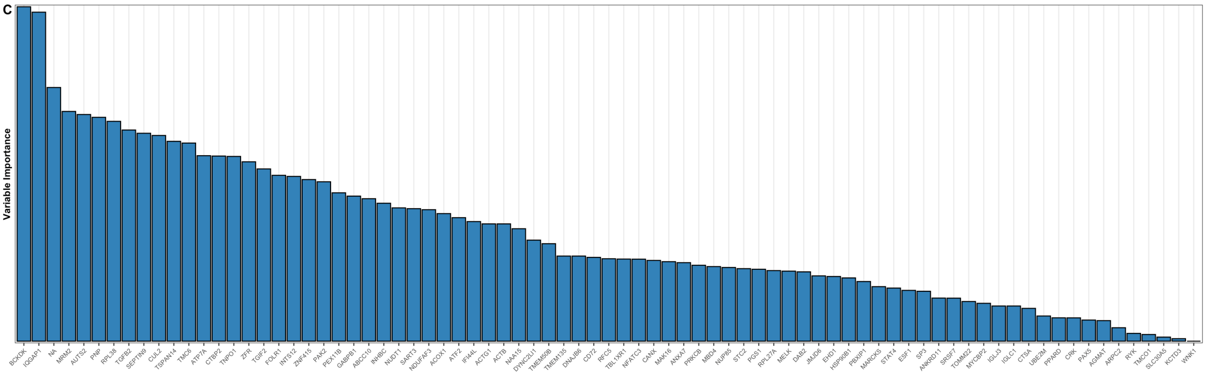

Supplementary Figure 1: A Results of the model optimization are provided in heatmap format. An elastic net generalized linear model optimized using repeated ten-fold cross-validation against a tuning grid of alpha values ranging from 0 to 1 (X-axis) and lambda values ranging from 0 to 0.1 (Y-axis). For each combination of alpha and lambda values, the cross-validated ROC value, sensitivity and specificity was recorded, and these results are provided in color-coded format according to the legend shown on the left. The optimal model with an alpha value of 0 and lambda value of 0.097 and a ROC value of 0.855. **B** Results of the application of the final model onto an independent set of test samples and the expression data set of preclinical models. **C** Variable importance plot with genes ranked according to their importance for accurate classification. The most important genes are shown to the left.

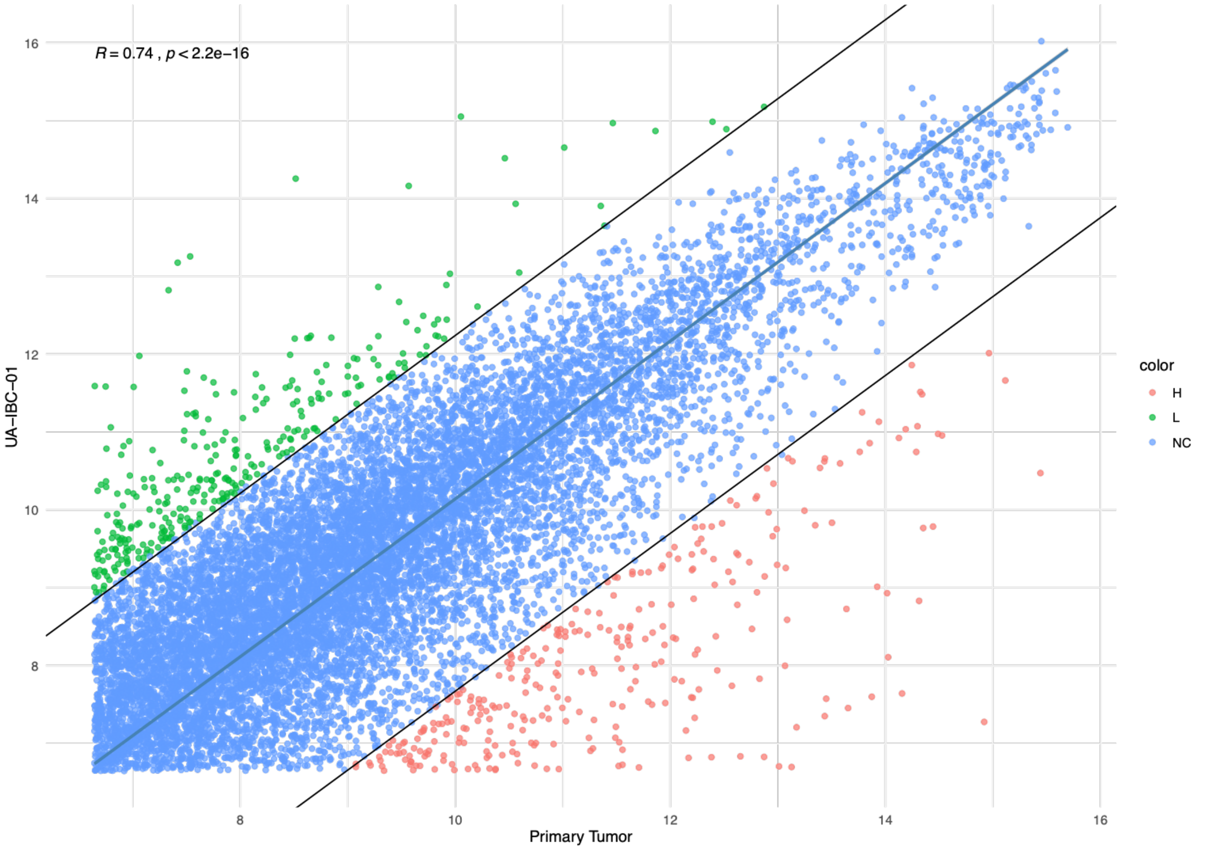

Supplementary Figure 2: Scatter plot showing genome-wide expression levels measured in the UA-IBC-01 cell line (Y-axis) in function of those measured in the corresponding primary tumor sample (X-axis). Regression line, correlation coefficient and boundaries for differential expression are indicated. Genes overexpressed in the primary tumor or in the UA-IBC-01 cell line are color-coded in red and green respectively, as shown in legend to the right of the plot (H=high in primary tumor; L=low in primary tumor; NC=no change).

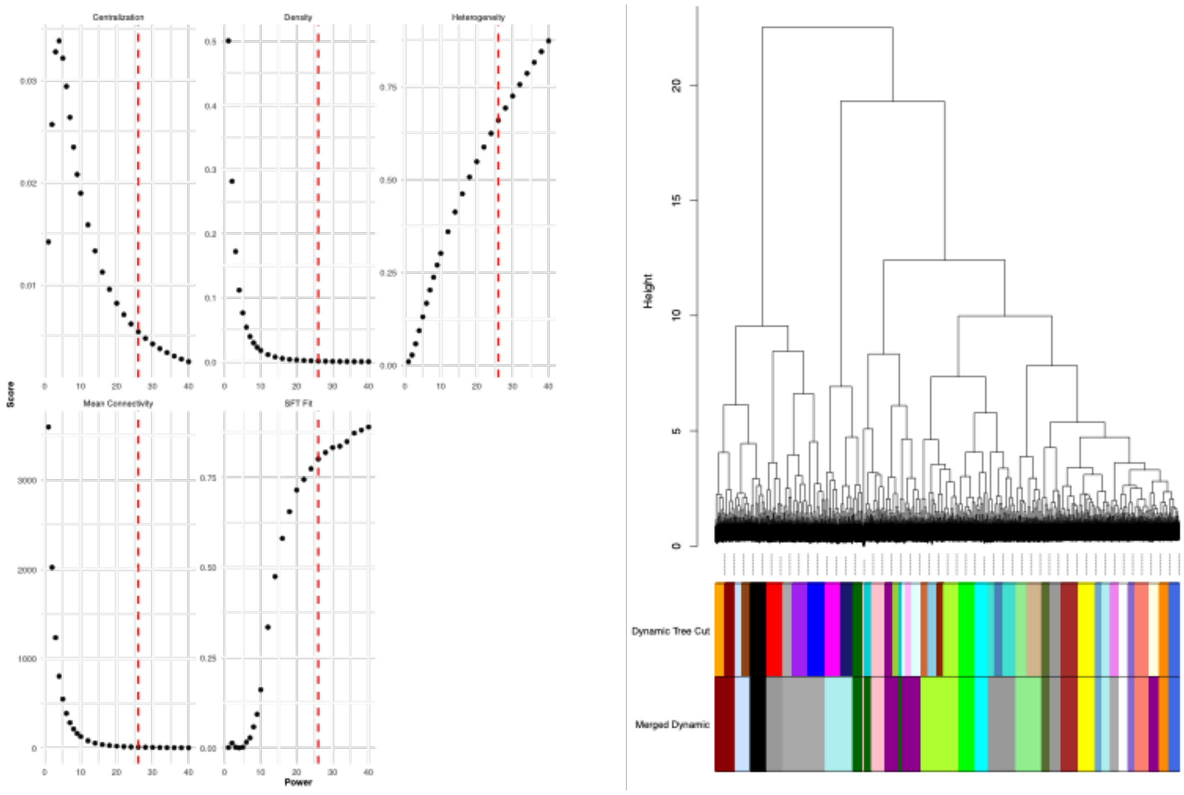

Supplementary Figure 3: A Topology parameters (Centralization, Density, Heterogeneity, Connectivity and Scale free Topology Fit) are provided for different soft threshold power values provided in the X-axis. The power value for which a scale free topology fit with an R square value of 0.80 was obtained to construct the coexpression network. This value (i.e. 26) is indicated by a red dashed vertical line. Other values are merely for reference. **B** Dendrogram resulting from Ward clustering on the topological overlap dissimilarity matrix used for identification of the co-expression clusters. Co-expression clusters are identified using a dynamic branch pruning algorithm and the resulting clusters are indicated in the first annotation track underneath the dendrogram using color-coded labels. Then, similar modules identified by co-clustering of their module eigengenes were merged resulting in the final set of co-expression modules, indicated in the second annotation track.

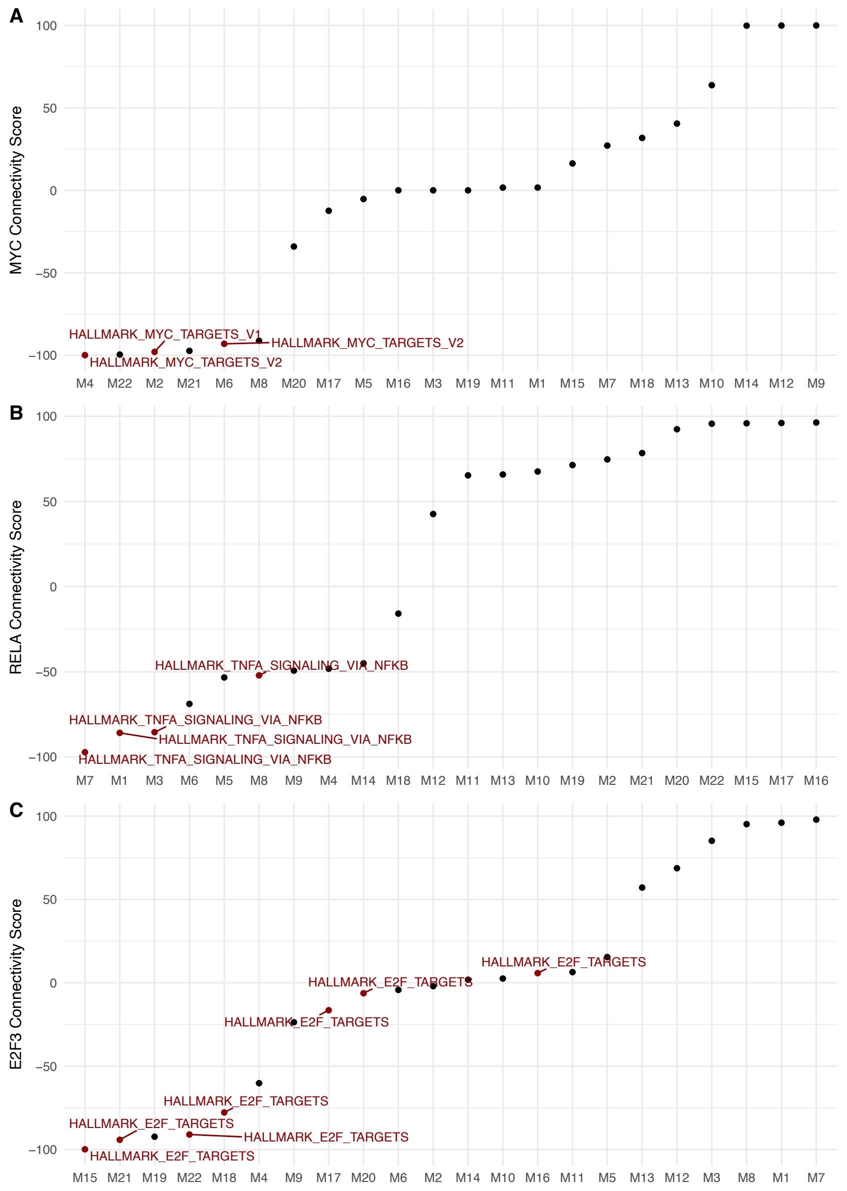

Supplementary Figure 4: The connectivity scores for MYC (**A**), RELA (**B**), and E2F3 (**C**) knockdown for each of the co-expression clusters are presented in scatter plot format. The clusters are shown along the X-axis in order of increasing connectivity score. The co-expression clusters enriched for MYC-target genes (**A**), NFkB target genes (**B**) and E2F target genes (**C**) are indicated using a label. From these plots it can be seen that the clusters associated with expression of these target genes show low connectivity scores for knockdown of the respective transcription factors.

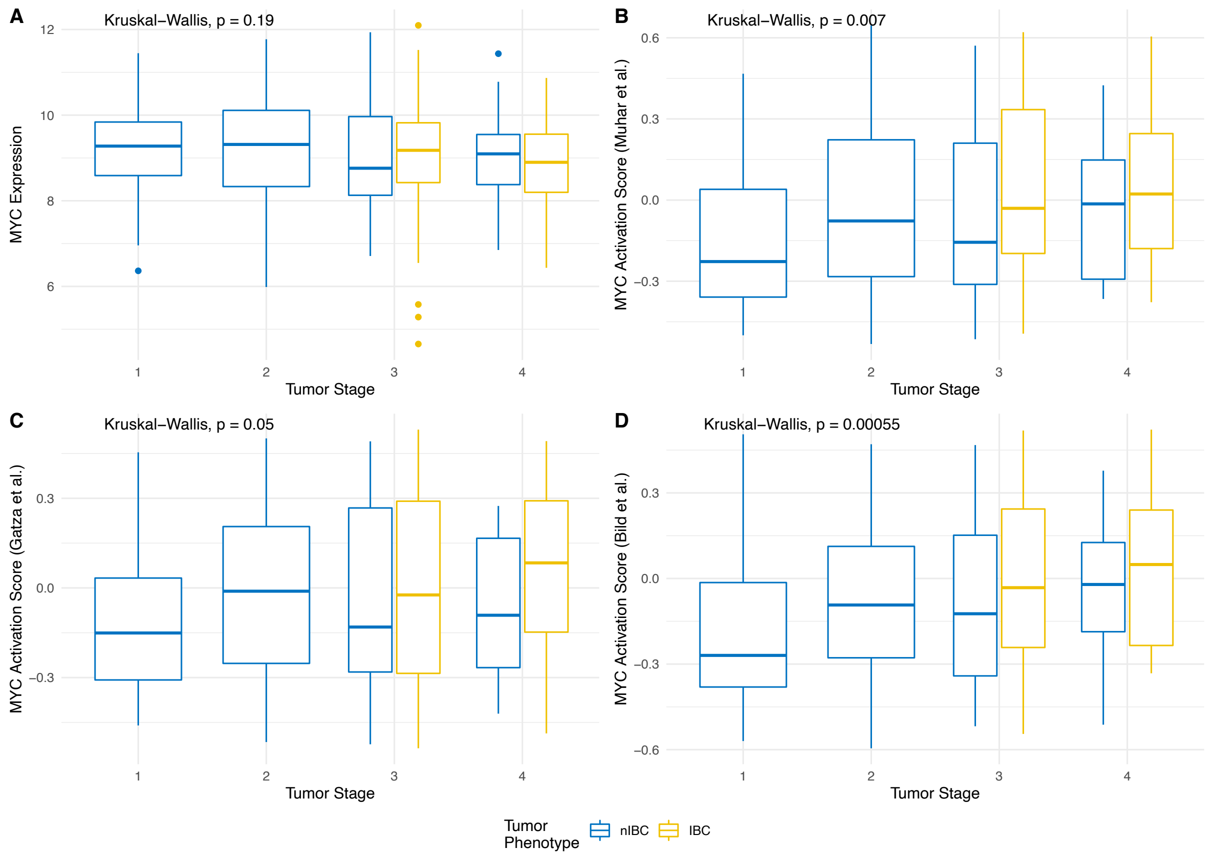

Supplementary Figure 5: A Boxplots showing MYC expression, MYC activation according to gene signature published by **B** Muhar et al., **C** Gatza et al., and **D** Bild et al. in function of tumor stage (X-axis). Boxplots corresponding to IBC and nIBC samples are color-coded shown in the legend underneath the figure. For IBC, only stage 3 or stage 4 tumor samples are available. P-values testing for significance of observed differences are indicated in the top left corner.

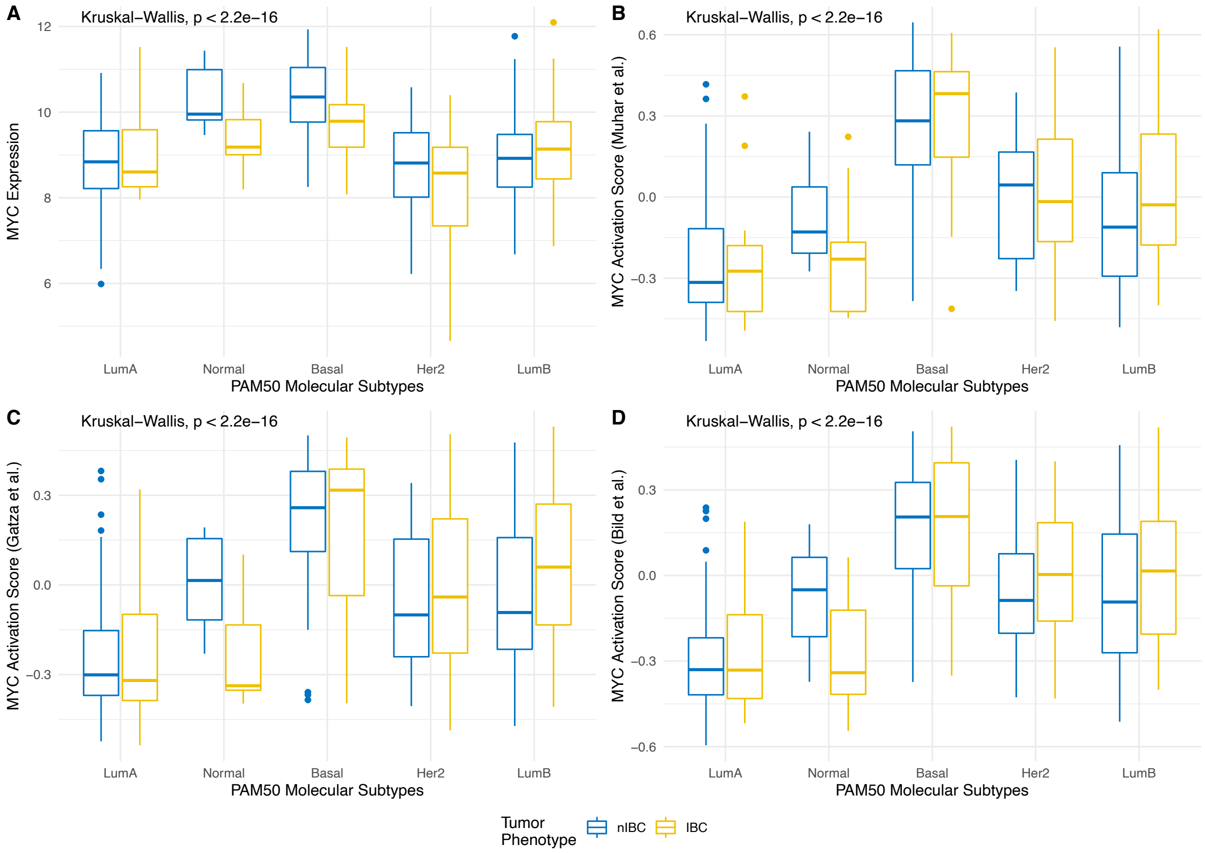

Supplementary Figure 6: **A** Boxplots showing MYC expression, MYC activation according to gene signature published by **B** Muhar et al., **C** Gatza et al., and **D** Bild et al. in function of the PAM50 subtypes (X-axis). Boxplots corresponding to IBC and nIBC samples are color-coded shown in the legend underneath the figure. P-values testing for significance of observed differences are indicated in the top left corner.

SUPPLEMENTARY TABLES

Supplementary Table 1: Gene set enrichment analysis to identify hallmark gene sets

Pathway	P	FDR	ES	NES	Size
HALLMARK_ADIPOGENESIS	0,891	0,963	-0,192	-0,803	140
HALLMARK_ALLOGRAFT_REJECTION	0,424	0,623	0,277	1,016	79
HALLMARK_ANDROGEN_RESPONSE	0,106	0,265	0,355	1,284	72
HALLMARK_ANGIOGENESIS	0,172	0,360	-0,451	-1,266	20
HALLMARK_APICAL_JUNCTION	0,117	0,265	0,323	1,244	107
HALLMARK_APICAL_SURFACE	0,522	0,687	-0,339	-0,967	22
HALLMARK_APOPTOSIS	0,107	0,265	-0,301	-1,225	117
HALLMARK_BILE_ACID_METABOLISM	0,204	0,364	-0,325	-1,167	53
HALLMARK_CHOLESTEROL_HOMEOSTASIS	0,460	0,658	-0,283	-0,999	51
HALLMARK_COAGULATION	0,041	0,169	-0,375	-1,417	70
HALLMARK_COMPLEMENT	0,211	0,364	0,296	1,141	109
HALLMARK_DNA_REPAIR	0,182	0,360	-0,283	-1,151	115
HALLMARK_E2F_TARGETS	0,191	0,360	-0,261	-1,136	174
HALLMARK_EPITHELIAL_MESENCHYMAL_TRANSITION	0,000	0,003	-0,421	-1,737	124
HALLMARK_ESTROGEN_RESPONSE_EARLY	0,000	0,003	-0,402	-1,724	161
HALLMARK_ESTROGEN_RESPONSE_LATE	0,001	0,010	-0,357	-1,531	161
HALLMARK_FATTY_ACID_METABOLISM	0,021	0,103	-0,338	-1,377	111
HALLMARK_G2M_CHECKPOINT	0,992	0,992	-0,168	-0,725	172
HALLMARK_GLYCOLYSIS	0,078	0,243	0,314	1,269	150
HALLMARK_HEDGEHOG_SIGNALING	0,474	0,659	0,380	1,000	17
HALLMARK_HEME_METABOLISM	0,317	0,511	-0,260	-1,077	130
HALLMARK_HYPOXIA	0,098	0,265	0,309	1,241	144
HALLMARK_IL2_STAT5_SIGNALING	0,046	0,178	0,356	1,379	111
HALLMARK_IL6_JAK_STAT3_SIGNALING	0,420	0,623	0,313	1,024	43
HALLMARK_INFLAMMATORY_RESPONSE	0,195	0,360	-0,303	-1,179	87
HALLMARK_INTERFERON_ALPHA_RESPONSE	0,113	0,265	0,371	1,296	58
HALLMARK_INTERFERON_GAMMA_RESPONSE	0,071	0,236	0,334	1,301	119
HALLMARK_KRAS_SIGNALING_DN	0,000	0,004	0,563	1,857	45
HALLMARK_KRAS_SIGNALING_UP	0,000	0,004	0,489	1,785	78
HALLMARK_MITOTIC_SPINDLE	0,701	0,846	-0,214	-0,910	156
HALLMARK_MTORC1_SIGNALING	0,859	0,955	0,199	0,829	175
HALLMARK_MYC_TARGETS_V1	0,194	0,360	0,270	1,133	185
HALLMARK_MYC_TARGETS_V2	0,002	0,010	0,501	1,709	52
HALLMARK_MYOGENESIS	0,011	0,063	-0,378	-1,468	87
HALLMARK_NOTCH_SIGNALING	0,711	0,846	0,286	0,817	24
HALLMARK_OXIDATIVE_PHOSPHORYLATION	0,906	0,963	-0,186	-0,808	174
HALLMARK_P53_PATHWAY	0,000	0,004	0,412	1,663	150
HALLMARK_PANCREAS_BETA_CELLS	0,667	0,833	0,353	0,837	11
HALLMARK_PEROXISOME	0,040	0,169	-0,372	-1,401	69
HALLMARK_PI3K_AKT_MTOR_SIGNALING	0,774	0,880	0,230	0,840	78
HALLMARK_PROTEIN_SECRETION	0,966	0,992	0,186	0,693	87
HALLMARK_REACTIVE_OXIGEN_SPECIES_PATHWAY	0,387	0,605	-0,318	-1,041	36
HALLMARK_SPERMATOGENESIS	0,761	0,880	-0,234	-0,838	53
HALLMARK_TGF_BETA_SIGNALING	0,493	0,666	0,295	0,982	47
HALLMARK_TNFA_SIGNALING_VIA_NFKB	0,061	0,216	0,325	1,306	144
HALLMARK_UNFOLDED_PROTEIN_RESPONSE	0,972	0,992	0,183	0,693	95
HALLMARK_UV_RESPONSE_DN	0,000	0,003	-0,435	-1,760	112
HALLMARK_UV_RESPONSE_UP	0,294	0,491	-0,264	-1,073	115
HALLMARK_WNT_BETA_CATENIN_SIGNALING	0,557	0,714	0,303	0,911	30
HALLMARK_XENOBIOTIC_METABOLISM	0,088	0,259	-0,301	-1,242	125

Supplementary Table 2: Regression analysis for each MYC-related feature

		MYC Expression			MYC Activation (Muhar et al.)			MYC Activation (Gatza et al.)			MYC Activation (Bild et al.)		
Model	*Term*	*Estimate*	*Standard Error*	*P-Value*	*Estimate*	*Standard Error*	*P-Value*	*Estimate*	*Standard Error*	*P-Value*	*Estimate*	*Standard Error*	*P-Value*
Original	(Intercept)	9,806	0,140	0,000	0,192	0,032	0,000	0,136	0,031	0,000	0,087	0,031	0,005
Original	IBC vs. nIBC	-0,833	0,196	0,000	-0,047	0,045	0,296	-0,052	0,044	0,230	-0,025	0,044	0,562
Original	ER moderate vs. ER low	-0,754	0,186	0,000	-0,340	0,043	0,000	-0,289	0,041	0,000	-0,272	0,041	0,000
Original	ER high vs. ER low	-0,931	0,180	0,000	-0,347	0,041	0,000	-0,234	0,040	0,000	-0,244	0,040	0,000
Original	IBC - ER moderate	0,599	0,283	0,035	0,068	0,065	0,299	0,084	0,063	0,182	0,064	0,063	0,310
Original	IBC - ER high	1,414	0,306	0,000	0,251	0,070	0,000	0,226	0,068	0,001	0,192	0,068	0,005
Block.Stage	(Intercept)	9,939	0,195	0,000	0,125	0,045	0,006	0,095	0,043	0,029	0,022	0,043	0,613
Block.Stage	IBC vs. nIBC	-0,697	0,221	0,002	-0,047	0,051	0,355	-0,037	0,049	0,450	-0,024	0,049	0,624
Block.Stage	ER moderate vs. ER low	-0,797	0,192	0,000	-0,326	0,044	0,000	-0,284	0,042	0,000	-0,258	0,042	0,000
Block.Stage	ER high vs. ER low	-0,948	0,182	0,000	-0,348	0,042	0,000	-0,239	0,040	0,000	-0,246	0,040	0,000
Block.Stage	IBC - ER moderate	0,630	0,294	0,033	0,043	0,067	0,526	0,070	0,065	0,281	0,043	0,065	0,510
Block.Stage	IBC - ER high	1,479	0,317	0,000	0,242	0,073	0,001	0,230	0,070	0,001	0,171	0,070	0,015
Block.Stage	Stage 2 vs. Stage 1	-0,038	0,185	0,836	0,098	0,042	0,020	0,075	0,041	0,069	0,086	0,041	0,036
Block.Stage	Stage 3 vs. Stage 1	-0,229	0,197	0,246	0,067	0,045	0,138	0,024	0,044	0,575	0,062	0,044	0,157
Block.Stage	Stage 4 vs. Stage 1	-0,474	0,238	0,047	0,081	0,054	0,137	0,048	0,053	0,361	0,134	0,053	0,011
Block.PAM50	(Intercept)	9,097	0,255	0,000	-0,141	0,059	0,017	-0,170	0,056	0,003	-0,211	0,057	0,000
Block.PAM50	IBC vs. nIBC	-0,430	0,184	0,020	0,014	0,042	0,740	0,005	0,041	0,906	0,016	0,041	0,698
Block.PAM50	ER moderate vs. ER low	-0,157	0,252	0,535	-0,102	0,058	0,077	-0,091	0,056	0,101	-0,086	0,056	0,130
Block.PAM50	ER high vs. ER low	-0,293	0,263	0,266	-0,129	0,060	0,034	-0,062	0,058	0,289	-0,086	0,059	0,142
Block.PAM50	IBC - ER moderate	0,045	0,266	0,866	-0,038	0,061	0,537	-0,022	0,059	0,709	-0,024	0,059	0,691
Block.PAM50	IBC - ER high	0,950	0,284	0,001	0,140	0,065	0,013	0,114	0,053	0,028	0,103	0,057	0,078
Block.PAM50	Normal-like vs. Luminal A	1,090	0,291	0,000	0,080	0,067	0,231	0,125	0,064	0,052	0,088	0,065	0,175
Block.PAM50	Basal vs. Luminal A	1,163	0,253	0,000	0,423	0,058	0,000	0,383	0,056	0,000	0,359	0,057	0,000
Block.PAM50	Her2 Enriched vs. Luminal A	-0,402	0,248	0,106	0,169	0,057	0,003	0,156	0,055	0,005	0,193	0,055	0,001
Block.PAM50	Luminal B vs. Luminal A	0,117	0,138	0,397	0,187	0,032	0,000	0,219	0,030	0,000	0,231	0,031	0,000

Supplementary Table 3: Clinical and pathological information of the patients (n=10) from whom the IBC models were derived

Model	Cell line/ Xenograft	ER status	PR status	HER2 status	Invasion/metastasis	Origin	Subtype primary tumor	Age at diagnosis	Lymph node status	Stage
SUM149	Cell line	Negative	Negative	Negative	Metastasis to multiple sites	Primary breast tumor	HR-			
SUM190	Cell line	Negative	Negative	Positive	Metastasis primarly to lung	Primary breast tumor	HR-			
KPL4	Cell line	Negative	Negative	Positive		Pleural effusion	HR+HER2+	52	Positive	
MDA-IBC3	Cell line/Xenograft	Negative	Negative	Positive	Metastasis to the lung	Pleural effusion	HR-HER2	53	Positive	
Mary-X	Xenograft	Negative	Negative	Negative	Metastasis primarly to lung, E-cadherin-positive emboli encircled by lymphatic vessels	Pleural effusion	HER2	45	Positive	
FC-IBC-01	Xenograft	Negative	Negative	Negative	E-cadherin-positive emboli encircled by lymphatic vessels	Pleural effusion	TNBC	47	Positive	IV
FC-IBC-02	Cell line/Xenograft	Negative	Negative	Negative	Metastasis to multiple sites, E-cadherin-positive emboli encircled by lymphatic vessels	Pleural effusion	TNBC	49	Positive	III
UA-IBC-01	Xenograft	Negative	Negative	Negative		Primary breast tumor	HR-HER2+			
TJ-IBC-04	Xenograft	Negative	Negative	Positive		Pleural effusion	HR+HER2+	32	Positive	IV
TJ-IBC-09	Xenograft	Negative	Negative	Negative		Pleural effusion	TNBC	43		IV
