# Supplementary material for: Comparative transcriptional analyses of preclinical models and patient samples reveal MYC and RELA driven expression patterns that define the molecular landscape of IBC
Source: NPJ Breast Cancer. 2022 Jan 18;8:12. doi: 10.1038/s41523-021-00379-6 (PMC8766434; doi:10.1038/s41523-021-00379-6)
Supplement: Supplementary file 5 — Reporting Summary [file 41523_2021_379_MOESM5_ESM.pdf]

nature portfolio

Corresponding author(s): Steven Van Laere, Charlotte Rypens, Francois Bertucci

Last updated by author(s): Jul 16, 2021

Reporting Summary

Nature Portfolio wishes to improve the reproducibility of the work that we publish. This form provides structure for consistency and transparency in reporting. For further information on Nature Portfolio policies, see our Editorial Policies and the Editorial Policy Checklist.

Statistics

For all statistical analyses, confirm that the following items are present in the figure legend, table legend, main text, or Methods section.

n/a	Confirmed	
☐	☒	The exact sample size (n) for each experimental group/condition, given as a discrete number and unit of measurement
☐	☒	A statement on whether measurements were taken from distinct samples or whether the same sample was measured repeatedly
☐	☒	The statistical test(s) used AND whether they are one- or two-sided<br>*Only common tests should be described solely by name; describe more complex techniques in the Methods section.*
☐	☒	A description of all covariates tested
☐	☒	A description of any assumptions or corrections, such as tests of normality and adjustment for multiple comparisons
☐	☒	A full description of the statistical parameters including central tendency (e.g. means) or other basic estimates (e.g. regression coefficient) AND variation (e.g. standard deviation) or associated estimates of uncertainty (e.g. confidence intervals)
☒	☐	For null hypothesis testing, the test statistic (e.g. F, t, r) with confidence intervals, effect sizes, degrees of freedom and P value noted<br>*Give P values as exact values whenever suitable.*
☒	☐	For Bayesian analysis, information on the choice of priors and Markov chain Monte Carlo settings
☒	☐	For hierarchical and complex designs, identification of the appropriate level for tests and full reporting of outcomes
☒	☐	Estimates of effect sizes (e.g. Cohen's d, Pearson's r), indicating how they were calculated

Our web collection on statistics for biologists contains articles on many of the points above.

Software and code

Policy information about availability of computer code

Data collection	No software was used for data collection.
Data analysis	All code generated in part of this publication is available at https://github.com/StevenVanLaere/IBCmodels. References towards software packages and versions are indicated throughout the manuscript.

For manuscripts utilizing custom algorithms or software that are central to the research but not yet described in published literature, software must be made available to editors and reviewers. We strongly encourage code deposition in a community repository (e.g. GitHub). See the Nature Portfolio guidelines for submitting code & software for further information.

Data

Policy information about availability of data

All manuscripts must include a data availability statement. This statement should provide the following information, where applicable:

- Accession codes, unique identifiers, or web links for publicly available datasets
- A description of any restrictions on data availability
- For clinical datasets or third party data, please ensure that the statement adheres to our policy

The data that support the findings of this study are available from the corresponding author upon reasonable request.

Field-specific reporting

Please select the one below that is the best fit for your research. If you are not sure, read the appropriate sections before making your selection.

☒ Life sciences ☐ Behavioural & social sciences ☐ Ecological, evolutionary & environmental sciences

For a reference copy of the document with all sections, see nature.com/documents/nr-reporting-summary-flat.pdf

Life sciences study design

All studies must disclose on these points even when the disclosure is negative.

Sample size	Sample size was not predetermined, because sample availability is a priori limited. In this manuscript, we describe the largest gene expression series ever described for both IBC preclinical models (N=10) and patient samples (N=146). Increasing the sample size beyound these limits is not feasible. However, based on previous calculations performed on publicaly availabe gene expression profiles from more than 1.000 patients classified according to ER status, we argue that sample sizes applied in this study are adequate. More precisely, using different series of ER+ and ER- breast tumor samples with variable sample sizes, we demonstrated that 40 breast cancer samples evenly distributed according to their ER status is sufficient to call at least 80% of the 2-fold expression differences significant at a P-value of 5%. For the cell line series, numbers included in present analyses are smaller (N=32), but the variance in the data set is arguably considerably lower. Therefore, it stands to reason that statistical power will only be mildly effected.
Data exclusions	No data were excluded.
Replication	For all but one of the cell lines, inclusion was based on the presence of triplicated gene expression data (technical replicates). For the clinical series, only one sample per patient was included. Both the cell line and patient series represent biological replicates for the conidtions of interest (i.e. IBC vs. nIBC). Where possible, randomization approaches comparing observed to expected distributions, with the latter being generated using at least 1.000 permutations were used or independent training and test sets for validation were generated.
Randomization	Not applicable.
Blinding	Not applicable.

Reporting for specific materials, systems and methods

We require information from authors about some types of materials, experimental systems and methods used in many studies. Here, indicate whether each material, system or method listed is relevant to your study. If you are not sure if a list item applies to your research, read the appropriate section before selecting a response.

Materials & experimental systems

n/a	Involved in the study	
☒	☐	Antibodies
☒	☐	Eukaryotic cell lines
☒	☐	Palaeontology and archaeology
☒	☐	Animals and other organisms
☒	☐	Human research participants
☒	☐	Clinical data
☒	☐	Dual use research of concern

Methods

n/a	Involved in the study	
☒	☐	ChIP-seq
☒	☐	Flow cytometry
☒	☐	MRI-based neuroimaging
